# Contexts and mechanisms relevant to General Practitioner (GP) based interventions to reduce adverse drug events (ADE) in community dwelling older adults: a rapid realist review

**DOI:** 10.12688/hrbopenres.13580.1

**Published:** 2022-07-21

**Authors:** Catherine Waldron, John Hughes, Emma Wallace, Caitriona Cahir, K. Bennett

**Affiliations:** 1 Data Science Centre, School of Population Health, RCSI University of Medicine and Health Sciences, Dublin 2, D02 DH60, Ireland; 2 Department of General Practice, University College Cork, Cork, Ireland; 3 Department of General Practice, RCSI University of Medicine and Health Sciences, Dublin 2, D02 DH60, Ireland

**Keywords:** Adverse drug events, Community dwelling, Older adult, General Practice

## Abstract

**Background:** Older adults in Ireland are at increased risk of adverse drug events (ADE) due, in part, to increasing rates of polypharmacy. Interventions to reduce ADE in community dwelling older adults (CDOA) have had limited success, therefore, new approaches are required.

A realist review uses a different lens to examine why and how interventions were supposed to work rather than if, they worked. A rapid realist review (RRR) is a more focused and accelerated version.

The aim of this RRR is to identify and examine the contexts and mechanisms that play a role in the outcomes relevant to reducing ADE in CDOA in the GP setting that could inform the development of interventions in Ireland.

**Methods:** Six candidate theories (CT) were developed, based on knowledge of the field and recent literature, in relation to how interventions are expected to work. These formed the search strategy. Eighty full texts from 633 abstracts were reviewed, of which 27 were included. Snowballing added a further five articles, relevant policy documents increased the total number to 45. Data were extracted relevant to the theories under iteratively developed sub-themes using NVivo software.

**Results:** Of the six theories, three theories, relating to GP engagement in interventions, relevance of health policy documents for older adults, and shared decision-making, provided data to guide future interventions to reduce ADEs for CDOA in an Irish setting. There was insufficient data for two theories, a third was rejected as existing barriers in the Irish setting made it impractical to use.

**Conclusions:** To improve the success of Irish GP based interventions to reduce ADEs for CDOA, interventions must be relevant and easily applied in practice, supported by national policy and be adequately resourced. Future research is required to test our theories within a newly developed intervention.

## Introduction

Adverse drug events (ADEs) are defined as ‘harmful unintended consequences of medication usage including medical errors, side effects, adverse drug reactions (ADR) and overdoses’ ^1, 2^. An alternative definition is “any untoward occurrence that may present during treatment with a pharmaceutical product but which does not necessarily have a causal relation to the treatment”, which acknowledges that not all events are necessarily drug-related and that it is not always possible to ascribe causality ^3^. Polypharmacy is most commonly defined as the concurrent use of ≥5 drugs ^4– 6^, which may contribute to increased levels of potentially inappropriate prescribing (PIP) ^7– 9^.

Older adults in Ireland are at risk of ADEs due in part to multimorbidity and the increasing rates of polypharmacy in this age group rising from 17.8% to 82.6% among those aged ≥65 years over the past 25 years ^10^. Rates of hospital admissions due to ADR in Ireland for this population were 8.8% of which 57% were considered potentially avoidable ^11^. These rates are similar to other international studies ^12^.

The likelihood of an ADE increases significantly with increasing exposure to PIP and contributes to the economic burden of healthcare ^9^. The number of repeat prescriptions and patients’ adherence to their medications have also been shown to be significantly associated with adverse health outcomes ^13, 14^.

Consideration of strategies that may reduce the risk of ADEs is important. These include regular review of patients’ medical conditions, medications, effects of treatment in conjunction with the use of World Health Organisation prescribing indicators, use of alert tools within electronic health records (EHR) and clear patient communication in relation to the benefits and risks and importance of correct adherence to the medication are all means used to prevent ADEs ^15^. Another factor that may increase the risk of ADEs include a previous history of ADEs so clear documentation of such events in the patient record is important. 

However, interventions to reduce the incidence of ADEs, or their causes, in older patients in primary care settings have had little or no success; a number of systematic reviews on the topic concluded that new approaches were required to reduce ADEs in older adults and patient‐related outcomes should be assessed ^15– 17^. In order to develop a better understanding of how, why, when, where and for whom these interventions are effective or not, a closer examination of the data is required. A realist approach is particularly suited to the synthesis of evidence about complex interventions as it uses a different lens to examine why and how the interventions are supposed to work rather than if, they worked ^18– 22^. Using a diverse range of evidence, including theoretical and empirical literature and involving key stakeholders in the process will increase the clarity and understanding of why an intervention succeeds or not.

Realist reviews aim to identify what it is about interventions that generate change (i.e., the mechanisms) and under which circumstances the mechanisms are triggered (i.e., the contexts), which result in changes in the behaviour of the participants of the intervention (i.e., the outcome). These three elements, i) context, ii) mechanism and iii) outcome, are presented together as a statement or theory which attempts to describe what needs to happen for an intervention to work ^23^. Mechanisms can be divided into resource mechanisms and reasoning mechanisms. Resource mechanisms tend to be more concrete i.e. environmental, organisational or political, while reasoning mechanisms are more invisible responses to the context i.e. trust or confidence. We have differentiated between the two in this review where possible. By differentiating between them, it is hoped that the necessary resources can be more clearly identified.

The products of realist reviews are theories, often produced in the form of “if …. then” statements developed from one or more Context Mechanism Outcome Configurations (CMOCs) found in the available data that outlines the individual or collective responses to intervention strategies and resources. This methodology is supported by methodological guidance, publication standards and training materials for realist reviews ^24^.

A rapid realist review (RRR) is a more focused and accelerated version of a full realist review which aims to produce theories in a time-sensitive way and that is useful to a specific audience about emerging issues, while preserving the core elements of realist methodology ^25^.

The aim of this RRR is to identify and examine the contexts and mechanisms that play a role in preventing or increasing outcomes relevant to the reduction of ADEs in community dwelling older adults (CDOA) in the general practice setting that could inform the development of a successful intervention in the Irish community setting. Specifically, we examined evidence in the literature in relation to:

1. The mechanisms that cause GPs and their community dwelling older patients to respond to interventions to reduce the incidence of ADEs.
2. The contexts believed to influence whether different mechanisms produce their intended outcomes.
3. The circumstances in which interventions in GP practices to reduce ADEs in community dwelling older adults are most likely to be effective.

## Methods

The protocol for this RRR was published on PROSPERO (CRD4202127757) in October 2021.

There are several stages to a RRR, some are similar to a conventional systematic review, but others are quite different ( Table 1) ^24^. As the process is an iterative one, the steps may not move in a linear fashion, at times steps were retraced or revisited.

**Table 1.** Stages in a Rapid Realist Review.

Stages in a Rapid Realist Review	
1.	Gathering an expert research team
2.	Developing the Candidate Theories (CTs)
3.	Reference Panel feedback and revision of the CTs
4.	Developing and undertaking the Search Strategy
5.	Screening, selecting and appraising articles
6.	Extraction of the data
7.	Analysis and synthesis of the findings

### 1. Gathering an expert research team

 The expert team included two authors from the 2020 systematic review of interventions to reduce ADE-related outcomes in older adults ^17^, with expertise in data science and population health (KB, CC), an academic general practitioner (EW), a healthcare professional with a background in Realist methodology (CW), a pharmacist and PhD student (JH), and an information specialist (PM).

### 2. Developing the Candidate Theories (CTs)

Candidate theories were developed in relation to how an intervention is expected to work based on the research teams’ assumptions, experience and knowledge of the field and on a brief search for evidence from recent literature. The research team developed eight candidate theories.

### 3. Reference Panel feedback and revision of the CTs

These candidate theories were reviewed by a reference panel of general practitioners (GP) in Ireland providing practical feedback using their local contextual knowledge. The reference panel completed an online survey on how well the theories were understood, how relevant they considered them and how feasible they would be to implement in an Irish setting (Extended Data File 1). The survey was emailed to academic GP colleagues, posted on Twitter with tags to GP organisations in Ireland and emailed to GP members of the Primary Care Clinical Trials Network Ireland group.

Details of the findings of the Reference Panel Survey are in Extended Data File 2. In summary, two of the eight theories were amalgamated, as they were judged to have overlapping themes: communication, providing information and shared decision-making. A theory relating to home visits was excluded as the respondents indicated that although home visits could be helpful, they were impractical in practice. The candidate theories were revised based on this feedback. The revised candidate theories are presented in Table 2. The six revised candidate theories formed the basis for the search strategy for the review.

**Table 2.** Revised candidate theories.

Revised Candidate Theories based on Expert Panel and Research Team Feedback (ranked in order of relevance)
1. **Engagement of GPs in interventions** To engage GPs in interventions to reduce the levels of ADE in CDOA, the competing demands on their time, the complexity of their patients, and the barriers to changing or de-prescribing medications, must be addressed.
2. **Health and clinical guidelines or policies** If guidelines or policies consider multi-morbidity, polypharmacy, and the commonly encountered adverse drug events that occur in older adults, then they will be more relevant to GPs who will be more likely to use them in practice thereby reducing the risk of ADE.
3. **Continuity of Care** When CDOA have continuity of care, they feel more understood and supported and have increased trust in their GP, the GP will be more familiar with their patient’s individual needs and confident when providing care thus improving medication management and reducing the risk of ADE.
4. **Health Information Technology** If Health Information Technology, including summary electronic care records and clinically useful medication alert systems are available to GPs and are easy to use then GPs will feel more supported, informed and confident when prescribing or changing medications, thereby reducing the risk of ADE.
5. **Shared Decision Making** When GPs communicate effectively, engage and support their patients and/ or carers in shared decision-making, there will be increased mutual trust and understanding about their illnesses and medications and patients will feel empowered, thereby reducing the risk of ADE.
6. **Collaboration with Pharmacists** When GPs and pharmacists in primary care work together when caring for CDOA with polypharmacy, GPs will feel more supported, aware and confident in relation to their patients’ individual needs resulting in more appropriate prescribing thereby reducing the risk of ADE.

### 4. Developing and undertaking the search strategy

Six electronic databases were searched (Ovid Medline, Embase, CINAHL, Web of Science, Cochrane Library and Lens). Four broad search strings were used, *older adult, adverse drug event, primary healthcare and community dwelling*. Details of the search terms and findings for each database are outlined in Extended Data File 3. Filters included a date limitation (Jan 2011 – September 2021) and articles in the English language only.

### 5. Screening, selecting and appraising of articles

The theories influenced the inclusion and exclusion criteria for the RRR search. A pilot screening of ten articles allowed some clarifications to be made to the inclusion and exclusion criteria. Conflicts were resolved by discussion and input from a third reviewer (KB). Table 3 outlines the broad inclusion and exclusion criteria used. A detailed inclusion and exclusion criteria document was prepared for the two reviewers (CW, JH) (Extended Data File 4). Following the screening by title and abstract, a full text reading of the included articles was undertaken by one reviewer (CW). A second reviewer (JH) reviewed 20% of the excluded articles.

**Table 3.** Broad inclusion and exclusion criteria.

Filters	Inclusion Criteria	Exclusion criteria:
Published 2011 – 2021. English Language.	**Participants**: GPs, their nurses, patients, their informal carers and community pharmacists **Setting**: GP practices and any linked setting i.e. community pharmacy or patients home. Focus on those countries where GPs have gate-keeping functions similar to Ireland unless the topics relate to human behaviour, support systems, beliefs, attitudes, opinion and perspectives that might be comparable to an Irish population. **Articles** irrespective of study design, opinion pieces, policy or protocol Articles related to ADE i.e. PIP, Potentially inappropriate medication (PIM), Deprescribing, Reporting ADRs and Polypharmacy, where the GP or their staff have a role to play.	Transitions from hospital Nursing home or institutional settings In-patient hospital settings Articles reporting prevalence only Studies about pharmacists or public health nurses that do not also include a role for GPs. Studies about public health nurses Studies about home visits Studies that use GP data only Studies focused on patients < 65 years old Articles not immediately accessible via our own library (RCSI) or on open access.

Quality assessment (QA) of realist data is considered under the headings of relevance, rigour and richness ^26, 27^, defined in Table 4. A scoring system was developed to rate the articles.

**Table 4.** Quality Assessment definitions and scoring system.

QA	Definition	Scoring System
**Relevance**	Does the article provide information of value to the review in relation to interventions to reduce ADEs in community dwelling older adults in primary care or the GPs or patients’ responses/reactions to the resources and opportunities provided by in the intervention?	0 = very poor 1= poor 2 = good 3 = very good.
**Rigour**	Are the sources or methods used to generate the relevant data credible and trustworthy?	
**Richness**	Relates to the level of theoretical and conceptual development detail provided in the articles. It is used as a means to identify articles of most value in a realist review. To score highly an article should provide sufficient details in relation to how the approach used was expected to work; documenting the process and explaining contextual factors that influenced implementation and/or outcomes.	0 = nothing of interest, not focused on the topic of interest 1 = limited data of interest, likely to appear in other articles 2 = limited data of interest, but quick to extract it and could add weight to findings 3 = some good quality data 4 = much valuable data.

Ten percent of the articles included for full text review had QA by two reviewers independently and disagreements were discussed and resolved (CW, JH). The refined QA process was applied by one reviewer (CW) to the remaining articles. Relevance, rigour and richness was scored for those articles that met all inclusion criteria at the full text stage review. Richness was reassessed at the data extraction stage. 

The richness assessment at full text reading ensured only those with the most potential for providing rich data were included in the RRR. Only those articles with a score of three or four for richness were included.

### 6. Extraction of the data

The included articles were imported into NVivo© and data extraction was carried out using this software by CW. Retroductive and abductive reasoning are used to make inferences in relation to how the data might be configured to explain how, why and in what context an intervention might work. The candidate theories formed the basis for the extraction process; sub themes, contexts, mechanisms, outcomes and some intervention details were extracted under a selection of codes. These codes provided an extraction template and were modified and developed throughout the process following familiarisation with the data. The final codebook can be found in Extended Data File 5.

## Results

**Figure 1.**
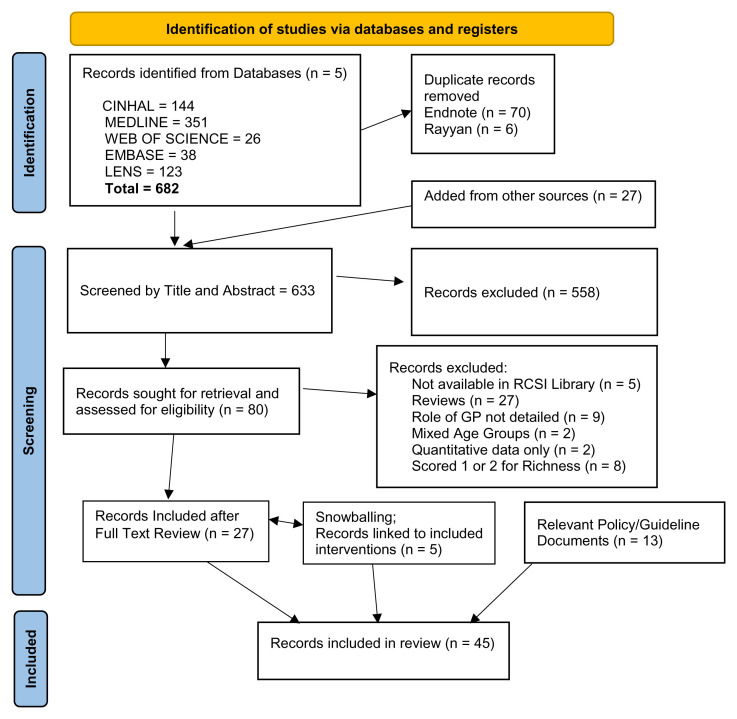
Prisma flow chart.

The details of the search and screening processes and findings are presented in the Prisma Flow Chart ( Figure 1). After removal of duplicates, 606 articles and an additional 27 articles from other sources were screened for relevance by one reviewer (N=633) (CW). Of these, 145 broadly relevant articles were more carefully screened independently by two reviewers (CW and JH). Eighty full texts were assessed for eligibility using the agreed inclusion and exclusion criteria (Extended Data File 5). Twenty-seven of these were included in the final review. Forward chasing added a further five articles reporting more details on already included interventions. In addition, a selection of Policy or Guidance documents on medication safety, polypharmacy or multimorbidity, which were identified by the team as being relevant to the review (N=13), were also included. The final number of articles/documents included in this RRR was 45 (Extended Data File 6).

Of the 45 articles, 13 were policy or guidance documents from Ireland, the UK and the World Health Organisation (WHO), published between 2015 and 2020 ^28– 40^, of which only two were specifically targeting older people ^35, 40^. The remaining 32 articles were published between 2007 and 2020 in Australia (N=5), Canada (N=1), Germany (N=7), Ireland (N=9), Switzerland (N=2), Thailand (N=1), The Netherlands (N=1), UK (N=3) and USA (N=3). Eleven articles reported on the design, implementation or evaluation of six different interventions. The remaining 21 articles were qualitative interviews, observational studies or expert opinion pieces.

Six articles included patients under 65 years as they were considered to contain relevant rich realist data. Of these, two included patients ≥18 years in relation to their experiences of adverse drug reactions ^41^ or polypharmacy ^42^, another three included patients ≥50 years ^43– 45^ and one included patients ≥60 years ^46^. Seven included patients who were ≥65 years ^47– 53^, five included patients who were ≥70 years ^54– 59^, three included patients who were ≥75 years ^60– 62^, one included patients who were ≥85 years ^63^. Of the remaining nine articles, four described the patients as living with multimorbidity ^64– 67^, four as elderly or old ^68– 71^ and one did not provide details ^72^.

### 7. Analysis and synthesis of the findings

Data were extracted relevant to the candidate theories under a series of iteratively developed sub-themes. The sub-themes, analysed as the interaction of specific contexts and mechanisms, were perceived to have a positive or negative impact on outcomes of relevance to reducing ADEs for CDOA in the GP setting. The analysis allowed the development of Context Mechanism Outcome Configurations (CMOCs) from the data to test the candidate theories to determine if they were supported, rejected or if any refinements were required based on the data. The resulting final theories are evidence-based, and intended to provide guidance in relation to the development of relevant interventions in the Irish GP setting. The CMOCs can be used to supplement their related theories in specific contexts. Each of the six theories, the analysis and related CMOCs are outlined below. Quotations from the articles to support the themes are presented in Table 5.

**Table 5.** Quotations from the articles to support the themes.

**Theory**	**Sub themes**	**Facilitators / Barriers**
T1: Engagement of GPs in interventions	Involving GPs in the Design and Implementation of Interventions	"Qualitative methods can contribute in several ways to the design and refinement of an intervention by identifying intervention components in need of further refinement, barriers or facilitators to implanting an intervention and involving users in the development process" **Clyne 2013** "Implementation research suggests that implementation programs should be tailored to individual barriers to introduce evidence-based knowledge into practice" **Jäger 2015** "Its application [The MRC Framework] ensured that the intervention was developed using the best available evidence, was acceptable to GPs and feasible to deliver in the clinical setting." **Clyne 2013**
		"Another issue was that they feared the use of a check- list aiming at standardizing or structuring the conversation would impede the individual care for the patient." **Jäger 2017.**
	Feedback and Support	"Some GPs appreciated that the implementation action plan helped them to raise awareness and to motivate the practice staff for change. ‘This helped us a lot. The motivation of the staff was stronger and as you can see we have realised most of the issues we have elaborated. That was most helpful (GP).’ ” **Jäger 2017b **
		"Less intensive feedback on prescribing behaviour is generally not sufficient to impact on prescribing practices" **Clyne 2016b**
	Research element	"Overall, patient identification and recruitment was reported as being quite onerous and was considered “…the only graft” (GP16, intervention practice) involved in participating in the study by many of the GPs." **Clyne 2016b**
	Time	“Practice nurses or other multidisciplinary team members can contribute in specific ways, including undertaking target assessment of chronic disease and psychological or functional capacity assessments that can support doctor and patient shared decision making." (Wallace 2015) "The risk patients come any way, at least once per year for the check-up (…) and I think you can combine this very well" **Jäger 2017**
		“Many chronic conditions, which were once managed in secondary care, have now become the remit of general practice. Adequate resourcing of primary care is of paramount importance in ensuring that this workload can be safely and effectively managed.” **ICGP** **2020**
	Case Complexity	"Respondents were also confident in their prescription of the identified PIMs and reported being comfortable with continued prescription." **Voight 2016**
		“I mean it [medication review] is time consuming which will be the biggest challenge… it’s nearly a bit of detective work going on, through the notes, trying to work out how did somebody on 16 items get onto some of these drugs.” P4 **Clyne 2013** “As was recognised by several physicians in the focus group discussion, risk is determined by more than just the crude number of medications being taken by a patient. A more nuanced approach to determining risk of ADR involves considering a combination of pharmacological, physiological and environmental determinants.” **Ridge 2019** Patient complexity (e.g. polypharmacy, multimorbidity), as well as prescriber complexity (e.g. multiple prescribers, poor communication, restricted autonomy) were all identified as factors contributing to a complex prescribing environment where PIP could occur **Clyne** **2016a**
	Isolation	"A key theme was GPs’ sense of professional isolation in the management of multimorbid patients. This emanated from the interplay between four aspects of the management of patients with multimorbidity: (i) the disorganisation and fragmentation of healthcare between primary and secondary care, (ii) the inadequacy of guidelines and evidence-based medicine for multimorbidity, (iii) challenges in delivering patient-centred, rather than disease-focused, care and (iv) barriers to shared decision-making." **Sinnott 2015b**
Theory 2: Health and clinical guidelines or policies	Relevance	To me, the guidelines are kind of a hindrance. At the moment they do not cater for older patients” **Schuling 2012.** “Although NICE full evidence summaries do provide information on the risks and benefits of treatment, few clinicians will have the time or expertise to read and interpret these documents, and the information is not consistently presented to facilitate comparison **.**” **Hughes 2013** “For example, GPs adopted a passive approach to medication management due to their uncertainty (lack of psychological capability) about which medications were most valuable in patients with multimorbidity, especially given the absence of satisfactory guidelines in this field” **Sinnott 2015b**
	Professional Judgement	“I don’t think that there is any good way to make that decision [regarding risks and benefits] other than your own clinical gut instinct or intuition.” **Fried 2011** “Although attempts are under way to improve the attentiveness of guidelines to multimorbidity, they will not be able to cover all eventualities in multimorbidity and some professional judgement will always be required” **Sinnott 2015a**
		“This leads to a situation where every individual recommendation made by a guideline may be rational and evidence based, but the sum of all recommendations in an individual is not.” **Wallace 2015**
Theory 3: Continuity of Care	Changes to practice	“Changes in the delivery of general practice service have reduced the provision of continuity of care. Patients value continuity, with over 80% of older patients (aged ≥75 years) in a recent UK survey reporting a preference for seeing a particular doctor in their general practice.” **Wallace 2015** “Our findings suggest that fragmentation of care between multiple prescribers results in poor communication of up-to-date patient medication information” **Clyne 2016a** “The communications space for shared care in community settings has receded and needs to be enlarged with concomitant improvements in communications technology, process and protocol to support effective multidisciplinary working.” **Rodgers 2014**
Theory 4: Health Information Technology	Information Technology	“Using the potential of information technology and data will help bridge the gaps between care services and enable people who use these services have access to their health care information, all of which can help optimise the use of medicines. **NICE Medicines** **optimisation 2016** Concomitant improvements in communications technology, process and protocol are urgently required to offset potentially serious risks to patient safety. **Rogers 2014**
	Alert Tools	“The use of a decision framework to identify (PIMs) for an individual could prove superior to lists of “drugs to avoid,” **Anderson 2020**.
		“In the interviews, the main barrier for using the tools was that they were not integrated into the practice software.” Jäger 2017b “To be effective, such tools need be applicable in routine clinical practice, not only in a research environment. However, as our study indicates, this gap may not have yet been successfully bridged in primary care.” Clyne 2016a “The answer to the problem does not seem to lie in mono-causal pharmaco-centered approaches or practical helps/tools.” Pohontsch-2017 "While some GPs appreciated the list, others had a more negative view, because they felt (severely) restricted in their freedom to choose medications. Rather than having a blacklist “banning” certain medications, they would prefer a whitelist indicating which medications can be safely used for elderly patients." Pohontsch 2017
Theory 5: Shared Decision Making (SDM)	Training and Skills in SDM	Training in shared decision-making could help GPs to elicit patient preferences. **Schuling** **2012** “The training helped the healthcare professionals understand how shared decision making differed from their current ways of working, by helping them improve their communication of risk and the way they explore what matters to patients. Some clinicians reported changing their view from we do this already to we could do this better.” **NICE 2019**
	Use of SDM	“The law now requires healthcare professionals to take reasonable care to ensure that the patient is aware of any material risks involved in any recommended treatment, and of any reasonable alternative or variant treatments.” **NICE 2019** “The qualitative evaluation of the pilot study indicated that GPs were very positive about both their experience and the patients’ feedback of the review process, and GPs were motivated to alter their prescribing practice: ‘O ya, and she was delighted, I stopped some of her other medications because she was in front of me and I had a bit of time to do it.’ P5.” **Clyne 2013**
		GPs also often criticize specialists’ lack of a holistic or geriatric view on elderly patients. Compared with the GP, they know much less about the patients concerning comorbidities, established medications or other specifics (e.g. medication sensitivity, changed metabolism) and may, therefore, consider risks and benefits less. Pohontsch 2017 “[GPs] feelings with regard to their management of the problem [Deprescribing] ranged from moderate optimism to something close to despair.” Schuling 2012
	Patient Knowledge/ Education	“… it’s [information leaflet] a good way of helping people, it’s a good negotiating thing, here’s the information…” P6” **Clyne 2013** “Patients should be empowered to manage their own health and be provided with the necessary skills and supports to do so.” **HSE Framework 2020**
		“Two factors were negatively associated with a good knowledge of the purpose of medications. The first factor was polypharmacy:... The second factor was receiving help with drug management: the more help, the lower the odds of good knowledge” **Hoisnard** **2018** “Almost none of our patients was able to use this tablet themselves. I think the medical assistant did it with them and read it to them or showed it to them (GP”) Jäger 2017 “When asked if they would have liked to receive the PILs, generally this patient group reported not having much use for such materials: ‘Oh no, no, I don’t welcome those sorts of things; they just pile up here in the house.’”. **Clyne 2016b** “They are not able to understand all this, I don’t even know if they understand me. If I would list all side effects (…) they would be very concerned (GP)”. **Jäger 2017b**
	Patient Preferences	“Their health goals focus more on quality of life than on extending their lives” **Schuliing** **2012**
Theory 6: Collaboration with Pharmacists	Respect Positive relationships Support	“In addition, most GPs work closely with a local pharmacist: the task perception of such pharmacists was an important factor when a GP was looking for decision support in medication review.” **Schuling 2012** “Good cooperation between pharmacists and GPs, and the willingness to share patient data were prerequisites.2 Geurts 2016 “The complexity of prescribing for the elderly is a lonely game.” (GP5) **Clyne 2016a**
	Workload	“Pharmacists are feeling the same pressure as physicians with regard to work volume and staffing.” **Chen 2017**
	The Irish Setting	Although enhanced communication between GPs and pharmacists is being investigated in other healthcare systems, it is not currently an option in Irish general practice due to the lack of community pharmacists. Sinnott **2015b**
		Many interventions to support medication review in primary care have used pharmacists (10–12). These interventions have shown inconsistent results and evidence of their impact on clinical outcomes is lacking. Furthermore, such approaches are not a pragmatic option in Irish healthcare where few publically funded community pharmacists exist. **Sinnott** **2017**

## Theory 1: Engagement of GPs with interventions

This theory focused on the challenges of engaging GPs with any planned intervention to reduce the levels of ADEs in CDOA. We hypothesised that the competing demands on their time, the complexity of their older patients, and the challenges associated with changing or deprescribing medications, must be addressed in order for the intervention to be accepted and implemented by them in a sustainable way.

### Facilitators

***Involving GPs in the design and implementation of interventions.*** It was generally accepted that involving GPs in the content and development of the intervention aided engagement and improved their practical implementation by identifying and minimising potential barriers ^45, 56, 67, 69, 73, 74^. The use of qualitative methods were thought to be useful at the development stage, many studies described focus groups and individual interviews with key stakeholders.

Having a plan that outlined how to implement an intervention helped. However, it was important that it should not impact excessively on existing processes in the practice or how GPs engage with their patients. Providing GPs with choices and options in relation to how they could adapt/tailor the intervention to suit their practice setting was highlighted.

***Feedback and support.*** Receiving follow-up support, seeing the benefits of their efforts, being given the opportunity to give and receive feedback were all seen as useful strategies in relation to successful implementation and sustainability of interventions.

### Challenges

***Research element.*** The research element of interventions resulted in additional pressures on GPs and the research team in relation to patient enrolment and consent.

Beyond the design and implementation of interventions, other contexts that challenged the success of interventions were identified; how these challenges could be overcome were suggested or applied in the interventions. Some of these contexts are outlined below.

***Time.*** A lack of time for medication review, for undertaking shared decision-making or for fully understanding the patient’s perspectives, was a consistent theme in most of the articles ^30, 32, 34, 39, 42– 47, 49, 51– 54, 56, 58, 63, 65– 67, 69, 72, 73, 75^. Facilitators to make the most of the GPs’ time were identified from these articles and are outlined in Table 6.

**Table 6.** Facilitators to making the most of GPs time.

Facilitators to making the most of GPs time
Making the most of existing opportunities to implement new processes, i.e. disease management programmes or yearly check-ups
Identifying the patients most at risk
Involving, delegating or expanding the role of other staff members (i.e. practice care assistants, receptionists, nurses)
Keeping the intervention simple
Keeping any training local, short, and in workshop style, with opportunities to share experiences
Practical, easy to use resources and tools
Use of technology/standardised software packages to record and share data

***Case complexity.*** A number of the studies reported that GPs are often not in agreement with each other or with the guidelines in relation to the medications that are appropriate to prescribe for older patients with multimorbidity. There was evidence that GPs acknowledged or recognised that potentially inappropriate prescribing occurs in practice ^43, 57^. However, regardless of whether this awareness of PIP was raised following an intervention, or already existed, GPs did not believe there was always a need for change. The GP's in-depth knowledge of the patient and their consideration of the importance of the GP-patient relationship supported their confidence in their individual decisions. GPs were confident about making "informed decisions" to use medications that had potentially adverse effects once these were balanced against treatment benefits, in the patient’s best interest ^53, 61, 72^. 

***Professional isolation.*** Professional isolation was identified as a factor for GPs. To address this, using existing relationships for example, informal peer support and relationships with Community Pharmacists could be means to providing support. Collaboration with Community Pharmacists and other GPs (which was the limited focus of this RRR), not only for their additional knowledge, but also to 'share the onus of responsibility', were identified as supportive strategies ^65, 67, 75^.

***Incentivisation.*** Structured medicines reviews (SMRs) with a focus on optimising appropriate polypharmacy may not be considered core work by Irish GPs ^56^. Other international studies also reflected this sentiment ^52, 72^. Reimbursement systems that include SMRs, which acknowledged the level of expertise and time involved, or external monitoring of patient medications by pharmacists, were identified as possible incentives ^56, 65^.

Other contexts that may influence the engagement of GPs in interventions that aim to reduce ADEs will be covered under the other more specific theories.

The data support Theory 1 and provide some individual Context – Mechanism - Outcome configurations (CMOCs) that could be applied to the design, implementation and sustainability of interventions to engage GPs in the reduction of ADEs in CDOAs in the Irish GP setting ( Table 7).

**Table 7.** Context-Mechanism-Outcome Configurations (CMOCs).

Context	Resource Mechanisms	Reasoning Mechanism	Outcomes
THEORY 1: Engagement of GPs in interventions			
Involving GPs in the design, content and Implementation of an Intervention to reduce ADEs	Practical to implement, feasible to deliver Provides relevant information	Involved, supported, listened to and respected. Altruism (feel good factor)	Increased participation and engagement in interventions Increased awareness of ADEs
Intervention champions within GP practices	Will raise awareness, remind and prompt action	GPs will feel motivated, encouraged and supported	Increased participation and engagement in interventions
Interventions that provide follow up support	GPs can give and receive feedback on their actions	GPs will feel encouraged, supported and involved	Sustainable interventions
Interventions that value the GPs time	Adapting existing processes to include MRs, Identifying Pts most at risk, expanding staff roles	GPs will feel less pressure, be more productive	Increased MRs and awareness of PIPs, reduction in ADE, increased staff involvement, decreased pressure on GPs time.
Interventions that include use of peer support (Other GPs and Community Pharmacists)	Reduced isolation, shared responsibility, added knowledge and expertise, articulation of issues and decision processes	GPs will feel supported, have increased confidence in their decisions	Increased collaboration, increased knowledge and awareness of PIPs, reduction in ADE
Interventions that include reimbursement or monitoring of MRs, PIPs or ADEs	Time and effort acknowledged Raise aware of the level of PIPs and ADEs	GPs will feel incentivised, motivated, respected, valued, reminded	Increased MRs, awareness of PIPs and reduction in ADE
THEORY 2 Health and clinical guidelines or policies			
Guidelines that address Multimorbidity and Polypharmacy	Move away from Single Disease Guidelines Reduce disease specific targets Prioritises pts QOL	Relevant Supported Informed Motivated	Guidelines applied in practice Reduced ADE Reduced Polypharmacy Improved Pt QOL
Guidelines that are clear and easy to use	Provide steps for implementation Summarise key points	Facilitated Accessible Activated	Guidelines applied in practice Reduced ADE
Guidelines that acknowledge the professional judgement of GPs	Address Multimorbidity and Polypharmacy Reduce disease specific targets Prioritises pts QOL	Respected, Competent Confident	Guidelines applied in practice Reduced ADE
Policies that adequately resource Medication Management	Audit Monitoring Reporting Incentivisation Funding	Encouraged Facilitated Aware Informed	Increased MR Reduced ADE
THEORY 5 Shared decision making			
GP Training in SDM	Practical skills Role play Online resources Support from colleagues Opportunity to challenge and discuss embedded attitudes Implementation strategies	Awareness Engagement Empowerment, Encouragement Motivation Confidence Competence Affirmation	Increased SDM Increased awareness of Patient preferences Increased MR Reduced ADE
Patient Training in SDM	Accessible Tailored User friendly	Awareness Engagement Empowerment Trust Confidence Listened to	Increased SDM Increased understanding Increased adherence to medications Reduced ADE
Policy	Government and Organisational support SDM is normal practice Funded Incentivisation	Supported Valued Motivated Facilitated	Increased SDM Increased MR Reduced ADE

## Theory 2: Health and clinical guidelines or policies

This theory considers the relevance of medication safety or prescribing policy or guidelines for older adults with multimorbidity or polypharmacy. We theorised that if guidelines or policy documents considered multimorbidity, polypharmacy, and the commonly encountered adverse drug events that occur in older adults, then they would be more relevant to GPs who would then be more likely to use them in practice thereby reducing the risk of ADEs.

### Relevance

Clinical guidelines tend to focus on single diseases and usually exclude older populations or those with multimorbidity. When guidelines do refer to older patients, they tend to be general statements about considering individual drug characteristics and age-adjusted doses of medications. Hughes et al in his 2013 review of UK NICE clinical guidelines stated that one of the major challenges facing clinical guidelines is accounting for multimorbidity ^64^. There was consensus in the articles reviewed that current clinical guidelines with their single disease focus act as a driver for polypharmacy in older multimorbid patients and do not providing guidance on how best to prioritise recommendations ^30, 58, 63, 64, 66, 71, 75^.

The lack of relevance of clinical guidelines for older patients with multimorbidity was shown to have negatively influenced the level of proactive involvement of GPs in relation to managing the medications of these patients ^67^.

However, more recently a NICE clinical guideline focusing on multimorbidity has highlighted issues to consider when caring for patients with multimorbidity and includes risk factors associated with the highest risk of experiencing ADEs. These patients could be targeted for structured medication reviews in general practice ^34^.

### Professional judgement

A number of articles addressed the dilemma for GPs when confronted with clinical guidelines that do not work for their patient ^53, 61, 63, 66, 69, 71, 72, 75^. The GPs used their judgement and accepted less stringent levels of disease control, stratified risk and benefits for individual diseases, prioritised the patients’ quality of life and preferences or modify guidelines in anticipation of adverse effects.

GPs felt justified in using their clinical judgement stating reasons such as the exclusion of older patients or those with multimorbidity in clinical trials underpinning clinical guidelines and the cumulative risk of polypharmacy if all guideline recommendations were followed. In addition, GPs used clinical and laboratory monitoring (e.g. blood tests, asking patients about side effects) as a means to support their decisions.

Sinnott *et al.* (2015) highlighted the relative autonomy experienced by GPs in the Irish healthcare system with respect to chronic disease management, which allowed them to use their judgement to a greater extent than GPs in other countries with tighter frameworks such as the UK ^66^.

### Using policy and guidelines in practice

Table 8 outlines what the data suggest in relation to the content of clinical guidelines and policy documents and the contexts that may encourage GPs caring for CDOA to use them in practice.

**Table 8.** Recommendations for contents and implementation of guideline and policy documents.

Recommendations for contents and implementation of guideline and policy documents	
a)	Be practical, useful and relevant
b)	Identify things that can be simply included in practice routines
c)	Provide clear information on the benefit/risk ratio of preventive medication for older patients
d)	Recognise the clinical judgment of GPs
e)	Reduce disease specific targets for multimorbid patients
f)	Provide financial remuneration or punitive measures, based on patient centred care
g)	Develop an action plan with the steps needed to put guidelines into practice.
h)	Set out additional costs or savings
i)	Identify a “practice champion” to motivate others to use the documents
j)	Carry out an assessment within the practice against the recommendations to identify gaps in current practice.
k)	Measure and record improvements and feedback this to staff and patients.

### Irish guidelines, policies and strategies

GPs in Ireland must follow the legislative requirements, local and national clinical guidelines, and professional standards when prescribing medications.

The Health Information and Quality Authority (HIQA) and The National Clinical Effectiveness Committee (NCEC) provide the framework for developing these clinical guidelines. The various disease specific guidelines provide Summary of recommendations and Summary of Good Practice Points documents, which are available to GPs, although they do not target them specifically; they acknowledge the professional judgement of GPs when the individual guideline recommendations are not appropriate or are declined by an individual patient.

The HIQA “Guidance on Medicines management” focuses predominately on patients in residential care, but is relevant to patients in a community setting ^28^. It recommends medicines management, which includes assessing, supplying, prescribing, dispensing, administering, reviewing and assisting people with their medicines, to reduce ADEs.

The Irish College of General Practitioners’ Quality and Safety in Practice Committee produces quick reference guidance, which can be accessed by their members. In “Medication Review – A Guide for GPs” they recommend that high-risk patients are identified and offered regular medication reviews. They also highlight the need for adequate resourcing to support the conduct of structured medication reviews in general practice ^30^.

The recently published National Framework for the Integrated Prevention and Management of Chronic Disease in Ireland 2020–2025 strategy is likely to have a significant impact on the workload of GPs in the community ^29^.

One element already being rolled out in Ireland is the Health Services Executive (HSE) Structured Chronic Disease Management (CDM) Programme, currently available to patients eligible for free GP care through a general medical services card who meet other criteria e.g. certain chronic medical conditions e.g. Type 2 diabetes, asthma, cardiovascular disease. A clinical data repository is proposed in the National Framework, that will gather information electronically on demographics, diagnoses, clinical examination results, diagnostic results and life-style risk factors on patients from GPs.

This National framework, if successfully implemented, goes some way to providing the elements outlined from the data in relation to the content of guidelines and the contexts that may encourage GPs caring for CDOA to use them in practice if they are properly resourced. To account for multimorbidity and polypharmacy, the theory might be amended to include respect for the GPs’ professional judgement, adequate resourcing to support the conduct of structured medication reviews, which are often complex and time-consuming and an electronic system of data collection and audit/feedback. The CMOCs for this theory are outlined in Table 6.

## Theory 3: Continuity of care

This theory focused on the relationship between the patient and the GP and continuity of care. It hypothesised that when CDOA have continuity of care, they feel more understood and supported and have increased trust in their GP, the GP will be more familiar with their patient’s individual needs and confident when providing care thus improving medication management and reducing the risk of ADEs.

A smaller number of articles addressed the issue of continuity of care; while they all acknowledged its importance, most also acknowledged the changing work environment that has reduced the level of continuity and increased the risk of fragmentation of care ^52, 55, 65, 73, 75^.

Improving the level and quality of communication between healthcare professionals and assigning a specific GP to patients identified as complex were the limited proposed solutions to this issue ^52^. The National Framework strategy aims to support improved sharing of patient health records and speaks about co-ordination of care but does not specifically refer to continuity of care.

In summary, while continuity of care was acknowledged as being important, there was insufficient evidence in the included articles to support or reject this theory.

## Theory 4: Health information technology

This theory focused on the use of health information technology (HIT) to manage medications. We also collected data in relation to the use of any other forms of technology likely to have an impact on patient care and ADEs.

We hypothesised that if HIT, including electronic summary care medical records and clinically useful medication alert systems, are available to GPs and are easy to use then GPs will feel more supported, informed and confident when prescribing or changing medications, thereby reducing the risk of ADEs.

### 4.1 Use of technology

Use of technology in general was felt to be a positive addition to improving medication safety, however the data related to its proposed use in the future rather than any current or past experience with it ^33, 52^.

### 4.2 Alert tools

Most of the data gathered from the included articles related to the use of medication alert systems ^42– 44, 47, 51– 54, 56, 61, 63, 67, 69, 70, 73^. When using an electronic alert system it was considered important to have it integrated into the general practice software for ease of use. Commonly handheld computers or tablets were used due to their mobility, robustness, simplicity of use, and low needs for technical support ^44, 70^. GPs used electronic and hardcopy versions of the tools either alone or together.

It was acknowledged that the alert tool needed to be specific to the country in which it was being used due to country-specific differences with respect to drug approval, prescribing practices, and treatment guidelines ^53, 54^. However, even when considerable effort was put into developing a specific tool for a specific setting, this did not guarantee successful implementation ^57^. Some level of frustration and confusion was expressed by researchers attempting to address the multiple barriers to their use, as their interventions failed to show impact ^57, 63^.

GPs were often sceptical in relation to the value of alert tools. Reasons for the scepticism included: tools not considering multimorbidity or polypharmacy, not designed for use with older patients, not easily used in daily practice, creating too many irrelevant alerts, too time consuming or not trusted to be up to date ^35, 44, 47, 53, 61, 70^.

Positive features that were identified or suggested in relation to making alert tools more acceptable in practice included: focusing on a select number of high-risk drugs, provision of clinical validation and intelligent alternative recommendations for alerts, allowing for clinical judgement, acknowledging the complexity of prescribing for older adults, allowing for patients’ preference, provision of training to ensure correct use of the system, understanding of its limitations and providing reminders to review existing medications or follow-up on pathology ^35, 51– 53, 56, 61, 65, 70, 73^.

In summary, there were limited data in relation to the use of electronic summary care records to reduce ADEs for CDOA. There was some evidence in relation to the use of alert tools. However, although a number of articles reported on interventions that had attempted to addresses the issues identified in our theory, accessibility and ease of use of alert tools, there appeared to be many other factors that needed to be taken into account. Training and building trust in the system, providing alternative recommendations, acknowledging the complexities and personal choices of older patients and the judgement of GPs are contexts and mechanisms that would need to be added to this theory and a new search for this specific data undertaken.

## Theory 5: Shared decision making

Shared decision-making (SDM) involves two-way communication between GPs and their patients. GPs share the best available evidence to patients and their carers so that they can consider all the options available to make informed decisions regarding their healthcare. It often includes medication review, deprescribing and discussions around adherence to recommendations ^37^.

This theory considered the contexts around SDM and what influence they have on its success. We examined evidence to support or reject the theory that if GPs communicate effectively, engage and support their patients and/ or carers in SDM, there will be increased mutual trust and understanding about their illnesses and medications and patients will feel empowered, thereby reducing the risk of ADEs. All but four of the included articles provide data relating to this theory ^28, 31, 47, 64^. The contexts relevant to GPs and patients are outlined separately.

### GPs perspectives 

***Training and skills in SDM.*** It was recognised that GPs required SDM training focusing on eliciting patient preferences, using decision-making tools, incorporating SDM into routine practice, communicating risks and benefits of treatments, broaching and discussing care plans, quality of life and end of life issues ^33, 37, 44, 53, 71^.

The types of training described included role-play, emphasis on practical skills, the opportunity to challenge and discuss embedded attitudes, online courses and the use of peer support ^37, 65, 67^.

***Facilitators.*** One facilitator identified to support the implementation of SDM in general practice included having it as a policy recommendation; making it routine practice, such as the “no decision about me, without me” strategy in the UK ^33^.

Providing GPs with the opportunities to experience SDM and receive affirmation of its usefulness and effectiveness had the potential to increase engagement in the process, provide empowerment, encouragement, motivation and build confidence and competence ^54^.

A positive relationship between the GP and their patient and the resulting mutual trust between them was identified by many as being a major facilitator for SDM ^55, 61, 62, 65, 66^.

The GP’s trust in the patient related to their belief in the accuracy of the information the patients communicated to them, in relation to symptoms, adherence etc. and the patient’s ability to understand the information they were sharing with them, fearing the information would only serve to confuse and worry them ^30, 40, 44^

***Challenges.*** The main challenge for implementing SDM identified in the articles was the time consuming element of SDM in an environment where consultation times were short, the workload extensive and, as already outlined in Theory 1 in relation to SMR, there was a lack of incentivisation ^30, 49, 54, 57^.

Some of the suggestions for overcoming this challenge included identifying those patients most at risk or adopting extended consultations for particularly complex patients ^30, 34, 38, 44, 51, 75^. The complexities of the patient, of prescribing and managing third party prescriptions, left GPs feeling overwhelmed, frustrated and lacking in confidence in relation to SDM, and its associated elements of medication review and deprescribing ^45, 49, 57, 58, 63^.

### Patient perspectives 

***Patient knowledge/education.*** The patient's awareness of and level of interest in their medications, how to use them and treatment options varied and was influenced by their level of polypharmacy and their need for the support of others ^50, 52, 59, 60^.

Methods to provide information to patients included information leaflets, use of technology; patient friendly interactive training tools or’ serious gaming’ approaches and posters to act as reminders about medication use. There were mixed results in relation to their use. One intervention using technology had referenced research supporting the use of iPads/tablets for patients with dementia but found the patients in their study were unable to use them ^43– 45^. Information leaflets were seen as useful by the GPs but not always valued by patients ^44, 54^.

Providing health education to patients so that they can play their role in SDM may require structural changes to the health services in relation to funding and access ^29, 37^. 

***Patient preferences.*** In general, patients regarded their Quality of Life (QoL) as being more important than mortality ^69^. Patients’ decisions regarding their treatment was influenced by their emotions, treatment goals and willingness to experiment. Patients’ willingness, and ability to be involved in decision making varied widely in the included articles ^29, 30, 32, 35, 37, 40, 54, 69, 71^.

This theory focused on the role of the GP in SDM process. However, the data suggest that for SDM to be successful, the role of the patient must be equally supported.

The theory should be amended thus; if GPs **and their patients and/ or carers** are supported to engage and communicate effectively in SDM, there will be increased mutual trust, empowerment, awareness and understanding about their illnesses and medications, thereby reducing the risk of ADEs. The CMOCs for this theory are outlined in Table 6.

## Theory 6: Collaboration with community pharmacists

This theory focused on collaboration with Community Pharmacists only. It proposed that when GPs and pharmacists in primary care work together when caring for CDOA with polypharmacy, GPs will feel more supported, aware and confident in relation to their patients’ individual health needs resulting in more appropriate prescribing, thereby reducing the risk of ADEs.

### Facilitators

***Respect.*** The data showed that, in general, GPs valued and respected the input of pharmacists for medication review or advice in relation to prescribing ^43, 62, 65, 70, 71, 75^. This existing positive perception is likely to enhance any collaboration between them.

***Supportive relationships.*** Developing and maintaining a positive relationship between the GP and the community pharmacist was regarded as an important prerequisite to collaboration, this included a willingness to share patient information. It was also identified as a means to reduce the sense of isolation felt by GPs and a way to share the responsibility of caring for complex patients ^56, 57, 62, 67^. This collaboration was perceived to facilitate patient centred care and medication management, and to reduce fragmentation of care ^43, 44, 46, 52, 62, 65, 73^.

***Patient trust.*** Patients were most likely to ask either their GP or their pharmacist if they needed information or had concerns about their medicines. They trusted and had generally positive relationships with their pharmacists ^41, 55^.

### Challenges

***Responsibility.*** A lack of clarity in relation to roles and responsibilities for medication management, particularly because of fragmentation or lack of continuity of care was reported in UK settings and identified as a potential gap in the integrated care process ^52^. Irish GPs have also indicated that they do not consider SMR to be their core responsibility ^56^. 

***Workload.*** The existing workload of community pharmacists may limit the role they can currently play ^49, 67^.

***Collaboration in the Irish setting.*** While existing Irish policy, guidance and strategy documents acknowledge the importance of collaboration in general and specifically between GPs and pharmacists, there is currently no formal arrangement and existing shortages of community pharmacists limit the impact of informal collaboration ^29, 31, 34, 67^. Research is required in the Irish setting to provide further evidence.

The data support this theory in relation to positive and respectful relationships and providing support and increased drug knowledge to GPs. However, a number of barriers must be surmounted to facilitate a more formal and structured approach to medicines management between GPs and pharmacists in the community in the Irish setting. There is a need to identify which HCP is responsible for conducting a structured medication review, with sufficient resourcing to support this, and improved communication and HIT must all be in place before this collaboration can be included in any future intervention to reduce ADEs for CDOA. For this reason, the theory was rejected by the team.

## Discussion

Three theories were supported by data ( Theory 1, Theory 2, and Theory 5), one theory was supported to some extent ( Theory 6) and two theories lacked sufficient data to make any firm conclusions ( Theory 3 and Theory 4). The theories, relating to GP engagement in interventions ( Theory 1), relevance of health and clinical guidelines or policies ( Theory T2), and shared decision-making ( Theory 5), provided some context-mechanism-outcome configurations that can be used to guide future interventions to reduce ADEs for CDOA in an Irish setting by highlighting the facilitators and barriers to success.

Involving GPs in the design and implementation of interventions that target them was identified as a facilitator by ensuring that the intervention was relevant and easy to apply in practice ( Theory 1). However, insufficient internal or external support in the form of health policy or legislation, funding, incentivisation, feedback and follow-up were likely to hamper success. A SR of Australian GPs involvement in interventions to improve patient management by Bernardes *et al.* (2019) reported on the challenges of engaging GPs in interventions and concluded similarly that communicating directly with GPs, exchanging ideas and ensuring that the topics were relevant and useful to them, improved their engagement. Feedback was also identified as useful ^76^. 

Clinical guidelines and policies that account for multimorbidity and polypharmacy, respect GPs’ professional judgement and that have adequately resourced and monitored recommendations, are more likely to be used by GPs ( Theory 2). Recent Irish policy and guidelines, such as the National Framework for the Integrated Prevention and Management of Chronic Disease in Ireland 2020–2025 strategy, if successfully implemented, could support these requirements. A number of other studies have identified challenges in relation to the use of clinical guidelines by GPs; mistrust, being overloaded with information, lack of respect for their expertise and loss of autonomy to make their own decisions, as barriers, but acknowledge that knowing recommended practice is helpful ^76, 77^. 

The role of both the GP and the patient in the SDM process must be equally supported for it to have an impact on reducing ADEs in the community ( Theory 5). The need for tailored training for both stakeholders to acquire the skills for this complex interaction was identified, as were the challenges already identified in Theory 1 and Theory 2. The complexity of learning and using the skills of SDM for both the healthcare professional and the patient has been the topic of a number of recent publications, these publications are likely to assist in understanding what is required to support GPs and their patients in this process ^78^.

**Figure 2.**
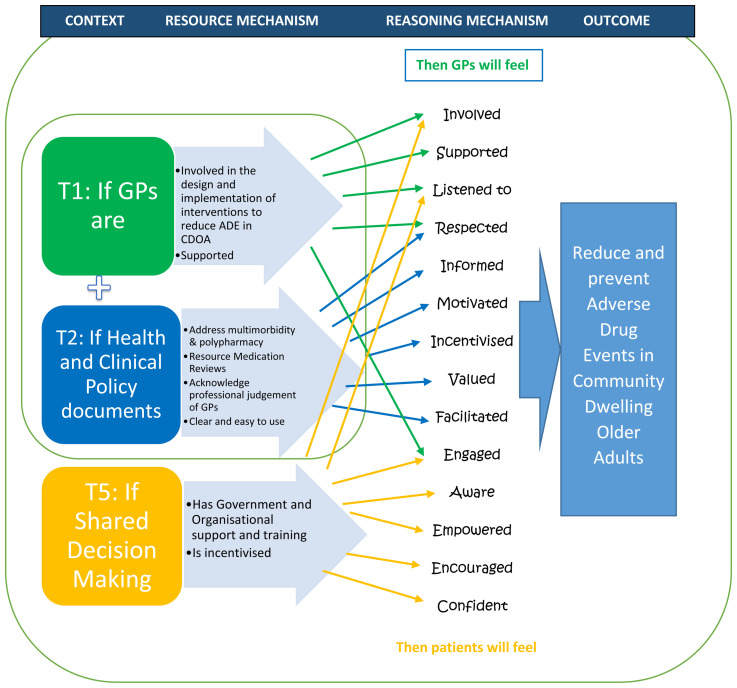
Summary “If..then” statements.

Theory 1 and Theory 2 are dependent on each other, Theory 5 could be tested independently, once the contexts identified in Theory 1 and Theory 2 are in place ( Figure 2).

Collaboration with community pharmacists was supported to some extent by the data ( Theory 6), but existing barriers in the Irish settings made it impractical. There is currently no formal arrangement in Ireland for this type of collaboration and existing shortages of community pharmacists limit the impact of informal collaboration. The role of clinical pharmacists as part of the primary care team, similar to those introduced in the UK, and tested in Ireland in a pilot study shows some promise ^79^.

There were insufficient data for two theories to make any firm conclusions ( Theory 3 and Theory 4). There were insufficient data in relation to the impact of continuity of care for either the patient or their GP in relation to ADEs ( Theory 3). In addition, the changing work environment for GPs in the community was highlighted, resulting in reduced levels of continuity of care.

Theory 4 focused on electronic care records and medication alert systems and evidence was in relation to the future rather than any current or past experience. There was some evidence in relation to the use of alert tools. Our theory focused on the accessibility and ease of use of alert tools; however, the data suggested that many other factors need to be taken into account including access to training, building trust in the system, providing alternative recommendations, acknowledging the complexities and personal choices of older patients and the judgement of GPs. A complete revision of this theory and new search for relevant data is proposed based on these findings.

## Strengths and limitations

The strength of this review is the additional insight it has provided in relation to past interventions, and their application specifically to an Irish context based on interventions limited to countries where GPs have gate-keeping functions. The date limitation (2011 – 2021) has also ensured the findings were relevant to current practice.

Limitations to this review are that the resulting final theories relied on the articles identified in our search; there was a lack of evidence to assess two theories. This may imply a lack of data on these topics or that a more in-depth search of the literature is required. The time limitations for this rapid review also meant that there was only one review for some of the screening and full text review of the articles. Quality appraisal was based on the research teams’ subjective judgements. In realist reviews, reasoning mechanisms are identified that might trigger positive or negative actions within the contexts. In reporting the CMOCs in this review, the mechanisms are presented in their positive form, but they could equally be working in their negative form if the resource mechanisms are not in place.

The range of outcomes measured in the interventions tended towards the conventional, i.e. changes to numbers of hospitalisations or medicines prescribed. Patient-reported and system-related outcomes provided greater opportunity to understand what works for whom and under what circumstances, how and why.

## Implications for practice

In order to improve the success of interventions to reduce ADEs for CDOA GPs must be involved in the design and implementation of the interventions to ensure their engagement. The interventions must be relevant and easily applied in practice, supported by national policy and be adequately resourced. In addition, tailored education for both GPs and patients is required to support SDM.

## Conclusion

Three theories with their related CMOCs, can be applied to the design and implementation of future interventions to reduce ADEs for CDOA in the Irish GP setting, based on this RRR. Future research is required to test our theories within an intervention.

## Data availability

### Underlying data

No data are associated with this article.

### Extended data

Zenodo: Realist-Review-of-ADEs-in-CDOA, https://doi.org/10.5281/zenodo.6803448 ^80^.

This project contains the following extended data:

- Extended Data File1: Reference Panel Survey.pdf
- Extended Data File 2: Results of the Reference Panel Survey.pdf
- Extended Data File 3: Search Strings for Six Databases and Findings.pdf
- Extended Data File 4: Inclusion and Exclusion Criteria Guidelines for Reviewers.pdf
- Extended Data File 5: Full text records sought for retrieval and assessed for eligibility
- Extended Data File 6: Included articles and policy documents with characteristics and quality assessment ratings
- Extended Data File 7: NVivo Code Book

### Reporting guidelines

Zenodo: Extended Data File 8: PRISMA checklist ‘Contexts and mechanisms relevant to General Practitioner (GP) based interventions to reduce adverse drug events (ADE) in community dwelling older adults; a rapid realist review’, https://doi.org/10.5281/zenodo.6803448 ^80^.

Data are available under the terms of the Creative Commons Attribution 4.0 International license (CC-BY 4.0).

## References

[1] Coleman JJ Pontefract SK : Adverse drug reactions. *Clin Med (Lond).* 2016;16(5):481–5. 10.7861/clinmedicine.16-5-481 27697815 PMC6297296

[2] European Directive 2010/84/EU of 15 December 2010 amending, as regards pharmacovigilance, Directive 2001/83/EC on the Community code relating to medicinal products for human use.2010. Reference Source

[3] Edwards IR Aronson JK : Adverse drug reactions: definitions, diagnosis, and management. *Lancet.* 2000;356(9237):1255–9. 10.1016/S0140-6736(00)02799-9 11072960

[4] Masnoon N Shakib S Kalisch-Ellett L : What is polypharmacy? A systematic review of definitions. *BMC Geriatr.* 2017;17(1):230. 10.1186/s12877-017-0621-2 29017448 PMC5635569

[5] Skinner M : A literature review: polypharmacy protocol for primary care. *Geriatr Nurs.* 2015;36(5):367–71.e4. 10.1016/j.gerinurse.2015.05.003 26122964

[6] Rankin A Cadogan CA Patterson SM : Interventions to improve the appropriate use of polypharmacy for older people. *Cochrane Database Syst Rev.* 2018;9(9):CD008165. 10.1002/14651858.CD008165.pub4 30175841 PMC6513645

[7] Galvin R Moriarty F Cousins G : Prevalence of potentially inappropriate prescribing and prescribing omissions in older Irish adults: findings from The Irish LongituDinal Study on Ageing study (TILDA). *Eur J Clin Pharmacol.* 2014;70(5):599–606. 10.1007/s00228-014-1651-8 24493365 PMC3978378

[8] Moriarty F Bennett K Cahir C : Potentially inappropriate prescribing according to STOPP and START and adverse outcomes in community-dwelling older people: a prospective cohort study. *Br J Clin Pharmacol.* 2016;82(3):849–57. 10.1111/bcp.12995 27136457 PMC5338119

[9] Cahir C Fahey T Teeling M : Potentially inappropriate prescribing and cost outcomes for older people: a national population study. *Br J Clin Pharmacol.* 2010;69(5):543–52. 10.1111/j.1365-2125.2010.03628.x 20573091 PMC2856056

[10] Moriarty F Hardy C Bennett K : Trends in polypharmacy and prescribing appropriateness from 1997 to 2012. *Int J Pharm Pract.* 2015;23:24–5. Reference Source

[11] Ahern F Sahm LJ Lynch D : Determining the frequency and preventability of adverse drug reaction-related admissions to an Irish University Hospital: a cross-sectional study. *Emerg Med J.* 2014;31(1):24–9. 10.1136/emermed-2012-201945 23389832

[12] van der Hooft CS Dieleman JP Siemes C : Adverse drug reaction-related hospitalisations: a population-based cohort study. *Pharmacoepidemiol Drug Saf.* 2008;17(4):365–71. 10.1002/pds.1565 18302300

[13] Cahir C Bennett K Teljeur C : PIH30 Potentially inappropriate prescribing and adverse health outcomes in community dwelling older patients. *Br J Clin Pharmacol.* 2014;77(1):201–10. 10.1111/bcp.12161 23711082 PMC3895361

[14] Cahir C Fahey T Teljeur C : PIH30 Potentially Inappropriate Prescribing and Adverse Health Outcomes in Community Dwelling Older Populations. *Value in Health.* 2012;15(7):A541. 10.1016/j.jval.2012.08.1902 PMC3895361 23711082

[15] Khalil H Bell B Chambers H : Professional, structural and organisational interventions in primary care for reducing medication errors. *Cochrane Database Syst Rev.* 2017;10(10):CD003942. 10.1002/14651858.CD003942.pub3 28977687 PMC6485628

[16] Cooper JA Cadogan CA Patterson SM : Interventions to improve the appropriate use of polypharmacy in older people: a Cochrane systematic review. *BMJ Open.* 2015;5(12):e009235. 10.1136/bmjopen-2015-009235 26656020 PMC4679890

[17] Tecklenborg S Byrne C Cahir C : Interventions to Reduce Adverse Drug Event-Related Outcomes in Older Adults: A Systematic Review and Meta-analysis. *Drugs Aging.* 2020;37(2):91–8. 10.1007/s40266-019-00738-w 31919801

[18] Pawson R : The Science of Evaluation: A Realist Manifesto.London: Sage Publications Ltd;2013. Reference Source

[19] Rycroft-Malone J McCormack B Hutchinson AM : Realist synthesis: illustrating the method for implementation research. *Implement Sci.* 2012;7(1):33. 10.1186/1748-5908-7-33 22515663 PMC3514310

[20] Pawson R : Evidence- based Policy: A realist perspective.1st ed. London: SAGE Publications Ltd.;2006. Reference Source

[21] Pawson R Greenhalgh T Harvey G : Realist review--a new method of systematic review designed for complex policy interventions. *J Health Serv Res Policy.* 2005;10 Suppl 1:21–34. 10.1258/1355819054308530 16053581

[22] Pawson R Greenhalgh T Harvey G : Realist synthesis - an introduction.2004. Reference Source

[23] Wong G : Making theory from knowledge syntheses useful for public health. *Int J Public Health.* 2018;63(5):555–6. 10.1007/s00038-018-1098-2 29632957

[24] Wong G Greenhalgh T Westhorp G : RAMESES publication standards: realist syntheses. *BMC Med.* 2013;11:21. 10.1186/1741-7015-11-21 23360677 PMC3558331

[25] Saul JE Willis CD Bitz J : A time-responsive tool for informing policy making: rapid realist review. *Implement Sci.* 2013;8(1):103. 10.1186/1748-5908-8-103 24007206 PMC3844485

[26] Wong G : Data Gathering in Realist Reviews: Looking for needles in haystacks.In: *Doing Realist Research.* London: SAGE Publications Ltd;2018;131–46. 10.4135/9781526451729.n9

[27] Booth A Harris J Croot E : Towards a methodology for cluster searching to provide conceptual and contextual "richness" for systematic reviews of complex interventions: case study (CLUSTER). *BMC Med Res Methodol.* 2013;13(1):118. 10.1186/1471-2288-13-118 24073615 PMC3819734

[28] Health Information and Quality Authority: Medicines Management Guidance.2015. Reference Source

[29] Health Service Executive: National Framework for the Integrated Prevention and Management of Chronic Disease in Ireland 2020-2025.Dublin: HSE;2020. Reference Source

[30] Irish College of General Practitioners Quick Reference Guide: Medication Review – A Guide for GPs.Dublin: ICGP;2020. Reference Source

[31] Medical Council and Pharmaceutical Society of Ireland: Safe Prescribing and Dispensing of Controlled Drugs.2017. Reference Source

[32] National Collaborating Centre for Primary Care (UK), National Institute for Health and Clinical Excellence: Guidance: Medicines adherence: involving patients in decisions about prescribed medicines and supporting adherence.2009. 21834197

[33] NICE Medicines and Prescribing Centre (UK), National Institute for Health and Care Excellence: Guidelines: Medicines optimisation: the safe and effective use of medicines to enable the best possible outcomes.2015. 26180890

[34] National Institute for Health and Care Excellence: Multimorbidity: clinical assessment and management. UK;2016. Reference Source

[35] National Institute for Health and Care Excellence: Medicines optimisation: adverse outcomes from potentially inappropriate prescribing in older people living in the community. *Medicines Evidence Commentary.* UK: NICE;2016.

[36] National Institute for Health and Care Excellence: Multimorbidity and polypharmacy Key therapeutic topic.UK,2017. Reference Source

[37] National Institute for Health and Care Excellence: Shared decision making. Key therapeutic topic.2019. Reference Source

[38] Scottish Government Polypharmacy Model of Care Group: Polypharmacy Guidance, Realistic Prescribing.2018. Reference Source

[39] World Health Organisation: Medication Without Harm - Global Patient Safety Challenge on Medication Safety.Geneva;2017. Reference Source

[40] Yorkshire and Humber AHSN Improvement Academy and Connected Yorkshire: Effectiveness Matters; Reducing harm from polypharmacy in older people.University of York: Centre for Reviews and Dissemination;2017. Reference Source

[41] Patsuree A Krska J Jarernsiripornkul N : Experiences relating to adverse drug reactions in the community: a cross-sectional survey among patients and the general public in Thailand. *Expert Opin Drug Saf.* 2016;15(3):287–95. 10.1517/14740338.2016.1135127 26750422

[42] Halli-Tierney AD Scarbrough C Carroll D : Polypharmacy: Evaluating Risks and Deprescribing. *Am Fam Physician.* 2019;100(1):32–8. 31259501

[43] Jäger C Freund T Steinhäuser J : Impact of a tailored program on the implementation of evidence-based recommendations for multimorbid patients with polypharmacy in primary care practices—results of a cluster-randomized controlled trial. *Implement Sci.* 2017;12(1):8. 10.1186/s13012-016-0535-y 28086976 PMC5237147

[44] Jäger C Steinhäuser J Freund T : A tailored programme to implement recommendations for multimorbid patients with polypharmacy in primary care practices—process evaluation of a cluster randomized trial. *Implement Sci.* 2017;12(1):31. 10.1186/s13012-017-0559-y 28264693 PMC5339959

[45] Jäger C Szecsenyi J Steinhäuser J : Design and delivery of a tailored intervention to implement recommendations for multimorbid patients receiving polypharmacy into primary care practices. *Biomed Res Int.* 2015;2015:938069. 10.1155/2015/938069 25685818 PMC4313053

[46] Geurts MM Stewart RE Brouwers JR : Implications of a clinical medication review and a pharmaceutical care plan of polypharmacy patients with a cardiovascular disorder. *Int J Clin Pharm.* 2016;38(4):808–15. 10.1007/s11096-016-0281-x 27052212 PMC4929171

[47] Köberlein J Gottschall M Czarnecki K : General practitioners' views on polypharmacy and its consequences for patient health care. *BMC Fam Pract.* 2013;14:119. 10.1186/1471-2296-14-119 23947640 PMC3751821

[48] Anderson K Stowasser D Freeman C : Prescriber barriers and enablers to minimising potentially inappropriate medications in adults: a systematic review and thematic synthesis. *BMJ Open.* 2014;4(12):e006544. 10.1136/bmjopen-2014-006544 25488097 PMC4265124

[49] Chen Z Buonanno A : Geriatric Polypharmacy: Two Physicians' Personal Perspectives. *Clin Geriatr Med.* 2017;33(2):283–8. 10.1016/j.cger.2017.01.008 28364996

[50] Hoisnard L Santos-Eggimann B Chauvin P : Do older adults know the purpose of their medications? A survey among community-dwelling people. *Eur J Clin Pharmacol.* 2019;75(2):255–63. 10.1007/s00228-018-2575-5 30334201

[51] Ridge A Macintyre K Kitsos A : Assessing risk of adverse drug reactions in the elderly: a feasibility study. *Int J Clin Pharm.* 2019;41(6):1483–90. 10.1007/s11096-019-00908-1 31564043

[52] Rogers S Martin G Rai G : Medicines management support to older people: understanding the context of systems failure. * BMJ Open.* 2014;4(7):e005302. 10.1136/bmjopen-2014-005302 25011989 PMC4120437

[53] Voigt K Gottschall M Köberlein-Neu J : Why do family doctors prescribe potentially inappropriate medication to elderly patients? *BMC Fam Pract.* 2016;17(1):93. 10.1186/s12875-016-0482-3 27449802 PMC4957869

[54] Clyne B Bradley MC Hughes CM : Addressing potentially inappropriate prescribing in older patients: development and pilot study of an intervention in primary care (the OPTI-SCRIPT study). *BMC Health Serv Res.* 2013;13(1):307. 10.1186/1472-6963-13-307 23941110 PMC3751793

[55] Clyne B Cooper JA Boland F : Beliefs about prescribed medication among older patients with polypharmacy: a mixed methods study in primary care. *Br J Gen Pract.* 2017;67(660):e507–e18. 10.3399/bjgp17X691073 28533200 PMC5540192

[56] Clyne B Cooper JA Hughes CM : A process evaluation of a cluster randomised trial to reduce potentially inappropriate prescribing in older people in primary care (OPTI-SCRIPT study). *Trials.* 2016;17(1):386. 10.1186/s13063-016-1513-z 27488272 PMC4973100

[57] Clyne B Cooper JA Hughes CM : ‘Potentially inappropriate or specifically appropriate?’ Qualitative evaluation of general practitioners views on prescribing, polypharmacy and potentially inappropriate prescribing in older people. * BMC Fam Pract.* 2016;17(1):109. 10.1186/s12875-016-0507-y 27515854 PMC4982127

[58] Rozsnyai Z Jungo KT Reeve E : What do older adults with multimorbidity and polypharmacy think about deprescribing? The LESS study - a primary care-based survey. *BMC Geriatr.* 2020;20(1):435. 10.1186/s12877-020-01843-x 33129274 PMC7602330

[59] Reed RL Isherwood L Ben-Tovim D : Why do older people with multi-morbidity experience unplanned hospital admissions from the community: a root cause analysis. * BMC Health Serv Res.* 2015;15:525. 10.1186/s12913-015-1170-z 26613614 PMC4662024

[60] Heser K Pohontsch NJ Scherer M : Perspective of elderly patients on chronic use of potentially inappropriate medication - Results of the qualitative CIM-TRIAD study. *PLoS One.* 2018;13(9):e0202068. 10.1371/journal.pone.0202068 30231027 PMC6145513

[61] Magin P Goode S Pond D : GPs, medications and older people: A qualitative study of general practitioners' approaches to potentially inappropriate medications in older people. *Australas J Ageing.* 2015;34(2):134–9. 10.1111/ajag.12150 24754489

[62] Peterson GM Naunton M Deeks LS : Practice pharmacists and the opportunity to support general practitioners in deprescribing in the older person. *J Pharm Pract Res.* 2018;48(2):183–5. 10.1002/jppr.1427

[63] Pohontsch NJ Heser K Löffler A : General practitioners' views on (long-term) prescription and use of problematic and potentially inappropriate medication for oldest-old patients-A qualitative interview study with GPs (CIM-TRIAD study). *BMC Fam Pract.* 2017;18(1):22. 10.1186/s12875-017-0595-3 28212616 PMC5395870

[64] Hughes LD McMurdo ME Guthrie B : Guidelines for people not for diseases: the challenges of applying UK clinical guidelines to people with multimorbidity. *Age Ageing.* 2013;42(1):62–9. 10.1093/ageing/afs100 22910303

[65] Sinnott C Byrne M Bradley CP : Improving medication management for patients with multimorbidity in primary care: a qualitative feasibility study of the MY COMRADE implementation intervention. *Pilot Feasibility Stud.* 2017;3(1):14. 10.1186/s40814-017-0129-8 28331631 PMC5357807

[66] Sinnott C Hugh SM Boyce MB : What to give the patient who has everything? A qualitative study of prescribing for multimorbidity in primary care. *Br J Gen Pract.* 2015;65(632):e184–91. 10.3399/bjgp15X684001 25733440 PMC4337307

[67] Sinnott C Mercer SW Payne RA : Improving medication management in multimorbidity: development of the MultimorbiditY COllaborative Medication Review And DEcision Making (MY COMRADE) intervention using the Behaviour Change Wheel. *Implement Sci.* 2015;10(1):132. 10.1186/s13012-015-0322-1 26404642 PMC4582886

[68] Wallis KA Elley CR Moyes S : Safer Prescribing and Care for the Elderly (SPACE): a pilot study in general practice. *BJGP Open.* 2018;2(3):bjgpopen18X101594. 10.3399/bjgpopen18X101594 30564727 PMC6189787

[69] Fried TR Tinetti ME Iannone L : Primary care clinicians' experiences with treatment decision making for older persons with multiple conditions. *Arch Intern Med.* 2011;171(1):75–80. 10.1001/archinternmed.2010.318 20837819 PMC3021478

[70] Mallet L Spinewine A Huang A : The challenge of managing drug interactions in elderly people. *Lancet.* 2007;370(9582):185–91. 10.1016/S0140-6736(07)61092-7 17630042

[71] Schuling J Gebben H Veehof LJ : Deprescribing medication in very elderly patients with multimorbidity: the view of Dutch GPs. A qualitative study. *BMC Fam Pract.* 2012;13(1):56–62. 10.1186/1471-2296-13-56 22697490 PMC3391990

[72] Cantrill JA Dowell J Roland M : Qualitative insights into general practitioners' views on the appropriateness of their long-term prescribing. *Int J Pharm Pract.* 2000;8(1):20–6. 10.1111/j.2042-7174.2000.tb00982.x

[73] Anderson K Freeman C Foster M : GP‐Led Deprescribing in Community‐Living Older Australians: An Exploratory Controlled Trial. *J Am Geriatr Soc.* 2020;68(2):403–10. 10.1111/jgs.16273 31792947

[74] Clyne B Smith SM Hughes CM : Sustained effectiveness of a multifaceted intervention to reduce potentially inappropriate prescribing in older patients in primary care (OPTI-SCRIPT study). *Implement Sci.* 2016;11(1):79. 10.1186/s13012-016-0442-2 27255504 PMC4890249

[75] Wallace E Salisbury C Guthrie B : Managing patients with multimorbidity in primary care. *BMJ.* 2015;350:h176. 10.1136/bmj.h176 25646760

[76] Bernardes CM Ratnasekera IU Kwon JH : Contemporary Educational Interventions for General Practitioners (GPs) in Primary Care Settings in Australia: A Systematic Literature Review. *Front Public Health.* 2019;7:176. 10.3389/fpubh.2019.00176 31316961 PMC6609323

[77] Clyne B Fitzgerald C Quinlan A : Interventions to Address Potentially Inappropriate Prescribing in Community‐Dwelling Older Adults: A Systematic Review of Randomized Controlled Trials. *J Am Geriatr Soc.* 2016;64(6):1210–22. 10.1111/jgs.14133 27321600

[78] Oerlemans AJM Knippenberg ML Olthuis GJ : Learning shared decision-making in clinical practice. *Patient Educ Couns.* 2021;104(5):1206–1212. 10.1016/j.pec.2020.09.034 33041158

[79] Cardwell K Smith SM Clyne B : Evaluation of the General Practice Pharmacist (GPP) intervention to optimise prescribing in Irish primary care: a non-randomised pilot study. *BMJ Open.* 2020;10(6):e035087. 10.1136/bmjopen-2019-035087 32595137 PMC7322285

[80] Waldron C : Contexts and mechanisms relevant to General Practitioner (GP) based interventions to reduce adverse drug events (ADE) in community dwelling older adults; a rapid realist review.[Dataset],2022. 10.5281/zenodo.6803448 PMC10811420 38283368

