# Supplementary material for: Contexts and mechanisms relevant to General Practitioner (GP) based interventions to reduce adverse drug events (ADE) in community dwelling older adults: a rapid realist review
Source: HRB Open Res. 2023 Dec 28;5:53. Originally published 2022 Jul 21. [Version 2] doi: 10.12688/hrbopenres.13580.2 (PMC10811420; doi:10.12688/hrbopenres.13580.2)
Supplement: Peer review history

# Reviewer report: Reviewer response for version 2

**DOI:** 10.21956/hrbopenres.15171.r37623

**Published:** 2024-01-25

**Authors:** Christiane Muth

**Affiliations:** 1 Bielefeld University, Bielefeld, Germany

The authors are to be congratulated for this excellent article. In addition, their responses provide a rich source for interested readers - the authors deserve great thanks for their thorough presentation.

Are the rationale for, and objectives of, the Systematic Review clearly stated?

Yes

Is the statistical analysis and its interpretation appropriate?

Not applicable

Are sufficient details of the methods and analysis provided to allow replication by others?

Partly

Are the conclusions drawn adequately supported by the results presented in the review?

Partly

Reviewer Expertise:

clinical decision support in chroninc diseases with multimorbidity and polypharmacy in general practice

I confirm that I have read this submission and believe that I have an appropriate level of expertise to confirm that it is of an acceptable scientific standard.

# Reviewer report: Reviewer response for version 1

**DOI:** 10.21956/hrbopenres.14833.r35047

**Published:** 2023-07-21

**Authors:** Christiane Muth

**Affiliations:** 1 Bielefeld University, Bielefeld, Germany

**Summary:** The authors present a rapid realist review (RRR) with the aim to identify and examine the contexts and mechanisms that play a role in reducing adverse drug events (ADE) in community dwelling older adults (CDOA) in the GP setting that could inform the development of interventions in Ireland.

 Six candidate theories in relation to how interventions are expected to work were based on knowledge of the field and recent literature including a feedback from a reference panel of seven Irish GPs formed the search strategy. In an iterative approach, screening and refining of inclusion and exclusion criteria took place resulting in a detailed operationalization of broad inclusion and exclusion criteria with regard to participants, setting, and objective. Restrictions were applied with publication date (2022 to 2021), publication language (English only) and accessibility (either open access or RCSI database availability). The selection of articles was conducted by one reviewer with a quality control of 20% of the excluded articles by a second reviewer. Quality assessment with regard to relevance, rigour and richness was refined after a rating of 10% of the included articles by two reviewers and conducted by one reviewer for all articles. Poor richness (scoring less than three points at a four-point Likert scale) led to the exclusion of an article. The extracted data on subthemes, contexts, mechanisms, outcomes and some intervention details were based on the candidate theories and linked to codes that allowed explaining how, why and in which context an intervention might work, which was also expressed through context-mechanism-outcome configurations (CMOCs).

 In total, 27 articles (resulting from the systematic search in six electronic databases) plus 18 documents achieved from additional (hand-)searches were included which provided rich data for 3/6 pre-specified theories (THEORY 1: Engagement of GPs in interventions, THEORY 2: Health and clinical guidelines or policies, and THEORY 5: Shared decision making), insufficient data for Continuity of care (theory 3) and Health information technology (theory 4) and only some data for a Collaboration with community pharmacists (theory 6). Theory 6 was rejected as existing barriers in the Irish setting made it impractical to use. The authors concluded that interventions need to be relevant, easy to apply in practice, supported by national policy and adequately resourced to improve the success of GP-based interventions to reduce ADEs in CDOA in Ireland.

**Peer review**: This article addresses and allows new insides into a highly relevant topic. The authors clearly stated objectives and rationale for their RRR and provided rich details of the methods in the main text and in additional web-supplements. The conclusions are in general adequately supported by the results presented in the review. The manuscript is well-written and concise.

 My major concern is about the usage of the term “theory”, as it is applied to models (such as Continuity of care), principles (such as Shared Decision Making), processes (such as the engagement of certain health professionals), decision support (such as guidelines), and tools (such as Health information technology), and what does it implies to the application of the realist review methods. For example, the following quotations at table 5 were assigned to health information technology (“theory 4”):

- “Using the potential of information technology and data will help bridge the gaps between care services and enable people who use these services have access to their health care information, all of which can help optimise the use of medicines. NICE Medicines optimisation 2016
- Concomitant improvements in communications technology, process and protocol are urgently required to offset potentially serious risks to patient safety. Rogers 2014”

It would be worth a discussion if quotations would provide data to support “theory 3” (continuity of care or more specifically, informational CoC). This example highlights, that some of the “theories” can be tools or components of others.

 It would perhaps help if the term "theory" were avoided and replaced by another, neutral term (eg, rationale for components or mechanism/mode of action?) and if the authors explained why they treat care models, principles, tools etc. equally and discuss the strengths and limitations of this approach.

 In addition, I have some minor amendments, which may help to improve the manuscript:

 1. Introduction, first para: the authors present two different definitions of ADEs. It would be helpful, if they would state, which definition they used.

 2. Inclusion/exclusion criteria:

- The inclusion criterion “Focus on those countries where GPs have gate-keeping functions similar to Ireland unless the topics relate to human behaviour, support systems, beliefs, attitudes, opinion and perspectives that might be comparable to an Irish population.” is difficult to understand. For example, German GPs don’t have a gate-keeping role. However, German studies formed a relevant body of included studies. Why did the authors include them (and why did they exclude others)?
- Why did the authors include “opinion pieces”?
- Figure 1: the box about records excluded shows “Reviews (27)”. It is not clear, why these reviews were excluded, as the publication type was not presented as an exclusion criterion per se.

3. Selection:

- It would be helpful to present the connection between the results and the supporting evidence. For example, table 5 presents a quotation about patient preferences (“Their health goals focus more on quality of life than on extending their lives” Schuliing 2012). The study by Schuliing was about the view of Dutch GPs. Readers could misinterpret the quotation as patient’s view (while patients’ preferences widely vary – between persons but also within persons over time, also in CDOA: eg, https://pubmed.ncbi.nlm.nih.gov/31843855/ ^1^, https://pubmed.ncbi.nlm.nih.gov/33383951/ ^2^).
  I’d suggest presenting a “baseline table” of included documents in the main text.
- Please check the supplemental documents for inconsistencies: eg. Amos et al. and Bregnhøj et al. were classified as included in Extended data File 5 but are not presented in the final list of included studies (Extended Data File 6).

4. Quality Assessment: In Figure 1, “records linked to included interventions (n=5)” and “relevant policy documents/guidelines (n=13)” seem to bypass the quality assessment, while they are partly assessed in Extended Data File 6 – please check for inconsistencies. Why is the quality assessment of the guidelines/policy documents “N/A”? What does mean the criterion “Richness at extraction”(in addition to “richness”) in Extended Data File 6?

 5. Discussion of limitations and implications: the authors name a few limitations with regard to their methods. How do the authors assess the effect of their restrictions (e.g. publication language and period, quality feature "richness" as exclusion criterion) and limited resources (screening, data extraction and coding could mostly be done by only one person, the panel survey could not be tested a priori) on the results? Would there be a need for further research to support the results of the RRR?

Are the rationale for, and objectives of, the Systematic Review clearly stated?

Yes

Is the statistical analysis and its interpretation appropriate?

Not applicable

Are sufficient details of the methods and analysis provided to allow replication by others?

Partly

Are the conclusions drawn adequately supported by the results presented in the review?

Partly

Reviewer Expertise:

clinical decision support in chroninc diseases with multimorbidity and polypharmacy in general practice

I confirm that I have read this submission and believe that I have an appropriate level of expertise to confirm that it is of an acceptable scientific standard, however I have significant reservations, as outlined above.

## References

[1] : Health-related preferences of older patients with multimorbidity: an evidence map. *BMJ Open* .2019;9(12) : 10.1136/bmjopen-2019-034485 e034485 10.1136/bmjopen-2019-034485 31843855 PMC6924802

[2] : End-of-Life Care Preferences of Older Patients with Multimorbidity: A Mixed Methods Systematic Review. *J Clin Med* .2020;10(1) : 10.3390/jcm10010091 10.3390/jcm10010091 33383951 PMC7795676

# Response

**Published:** 2023-12-09

**Authors:** Catherine Waldron

**Affiliations:** Royal College of Surgeons in Ireland, Ireland

**Response to reviewer 2 Christiane Muth, Bielefeld University, Bielefeld, Germany**

 Thank you for taking the time to review our article and providing very helpful feedback.

 We will address each of your comments in the order you listed them:

 1. My major concern is about the usage of the term “theory”, as it is applied .to models (such as Continuity of care), principles (such as Shared Decision Making), processes (such as the engagement of certain health professionals), decision support (such as guidelines), and tools (such as Health information technology), and what does it implies to the application of the realist review methods. For example, the following quotations at table 5 were assigned to health information technology (“theory 4”):

 •             “Using the potential of information technology and data will help bridge the gaps between care services and enable people who use these services have access to their health care information, all of which can help optimise the use of medicines. NICE Medicines optimisation 2016

 •             Concomitant improvements in communications technology, process and protocol are urgently required to offset potentially serious risks to patient safety. Rogers 2014”

 It would be worth a discussion if quotations would provide data to support “theory 3” (continuity of care or more specifically, informational CoC). This example highlights, that some of the “theories” can be tools or components of others.

 It would perhaps help if the term "theory" were avoided and replaced by another, neutral term (eg, rationale for components or mechanism/mode of action?) and if the authors explained why they treat care models, principles, tools etc. equally and discuss the strengths and limitations of this approach.

 Thank you for your thoughts on the use of the term “theory” in our rapid realist review. The term theory when used in realist methodology refers to informal theory and is not the same as formal theory. We have stated in the introduction that the products of realist reviews are called “theories”,  often in the form of “if …. then” statements, developed from the Contexts, Mechanisms and Outcomes found in the available data that outlines the individual or collective responses to intervention strategies and resources. The “theory” therefore in realist reviews is an attempt to describe what needs to happen for an intervention to work. We have added an additional sentence to further explain what a realist theory is *“In order for it to be a realist theory, it must refer to the context and mechanisms and how they relate to outcomes (Hunter et al 2022.”*

 Wong in his article “Making theory from knowledge syntheses useful for public health. Int J Public Health. 2018;63(5):555–6.” states that there are many definitions of theory, with a simple definition being an attempt to organize the facts and that a theory must be supported by facts or data.

 The two quotations you picked out from table 5 in relation to Theory 4 were examples from the included articles to support that theory which was focused on the use of health information technology to manage medications and stated “If Health Information Technology, including summary electronic care records and clinically useful medication alert systems are available to GPs and are easy to use then GPs will feel more supported, informed and confident when prescribing or changing medications, thereby reducing the risk of ADE.” Theory 3 was about continuity of care and stated, “When community dwelling older adults have continuity of care, they feel more understood and supported and have increased trust in their GP, the GP will be more familiar with their patient’s individual needs and confident when providing care thus improving medication management and reducing the risk of ADE”. It was focused on the relationship between the patient and did not include the use of technology. Although it is possible that the same data can be used to support more than one theory, we don’t see that this is the case in this instance.

 As realist theories are the cornerstone of realist reviews, we do not see this as a limitation, but do acknowledge that there are some limitations to this rapid realist review, as there was a lack of data to assess two of the theories put forward.

 2. Introduction, first para: the authors present two different definitions of ADEs. It would be helpful, if they would state, which definition they used.

 Thank you for highlighting this, our intention was to use as wide a definition of ADEs as possible when screening the articles, we have added a line to explain this. This paragraph will now read:

*Adverse drug events (ADEs) are defined as ‘harmful unintended consequences of medication usage including medical errors, side effects, adverse drug reactions (ADR) and overdoses’ ^1,2^. An alternative definition is “any untoward occurrence that may present during treatment with a pharmaceutical product but which does not necessarily have a causal relation to the treatment”, which acknowledges that not all events are necessarily drug-related and that it is not always possible to ascribe causality ^3^. We accepted any definition of ADEs in this review.*

 3. Inclusion/exclusion criteria:

 The inclusion criterion “Focus on those countries where GPs have gate-keeping functions similar to Ireland unless the topics relate to human behaviour, support systems, beliefs, attitudes, opinion and perspectives that might be comparable to an Irish population.” is difficult to understand. For example, German GPs don’t have a gate-keeping role. However, German studies formed a relevant body of included studies. Why did the authors include them (and why did they exclude others)?

 We did allow for the inclusion of articles from countries with GPs with non-gate keeping functions if the topics relate to human behaviour, support systems, beliefs, attitudes, opinion and perspectives that might be comparable to an Irish population. We also outlined in Extended Data file 4 more details in relation to the inclusion and exclusion criteria. Although we focused on those countries where GPs have gate-keeping functions similar to Ireland: which included UK, Australia, New Zealand and the Netherlands, we also included Canada and USA, if the focus of the article was on family practice only and studies from other countries if the topics relate to human behaviour, support systems, beliefs, attitudes, opinion and perspectives that might be comparable to an Irish population.

 4. Why did the authors include “opinion pieces”?

 This is normal practice for realist reviews, as the perspectives of informed individuals may be a rich source of relevant data.

 5. Figure 1: the box about records excluded shows “Reviews (27)”. It is not clear, why these reviews were excluded, as the publication type was not presented as an exclusion criterion per se.

 As review articles, they may have included some of the individual articles already included in this review. Review articles also tend to summarise the findings, whereas we were most interested in the individual findings from each article. We did clarify in Extended data file 4 that relevant Systematic, Scoping or Literature Reviews would be included at the title and abstract screening stage and tagged as “Review Articles” and assessed at this stage for studies of interest.

 6. Selection: It would be helpful to present the connection between the results and the supporting evidence. For example, table 5 presents a quotation about patient preferences (“Their health goals focus more on quality of life than on extending their lives” Schuliing 2012). The study by Schuliing was about the view of Dutch GPs. Readers could misinterpret the quotation as patient’s view (while patients’ preferences widely vary – between persons but also within persons over time, also in CDOA: eg, https://pubmed.ncbi.nlm.nih.gov/31843855/1, https://pubmed.ncbi.nlm.nih.gov/33383951/2).

 This quotation related to the theory about shared decision making which stated, “When GPs communicate effectively, engage and support their patients and/ or carers in shared decision-making, there will be increased mutual trust and understanding about their illnesses and medications and patients will feel empowered, thereby reducing the risk of ADE”. All of the quotations in this section relate to GPs, but this one came under the heading of patient preferences, and as you say could have been clearer that it was the opinion of the GPs. We have added a clarification as we have done in some other quotations with [ ] brackets.

*“Their [the patients’] health goals focus more on quality of life than on extending their lives” Schuliing 2012*

 7. I’d suggest presenting a “baseline table” of included documents in the main text.

 We were limited to the number of tables we could include in the main text, and so provided these details in the extended data files.

 8. Please check the supplemental documents for inconsistencies: eg. Amos et al. and Bregnhøj et al. were classified as included in Extended data File 5 but are not presented in the final list of included studies (Extended Data File 6).

 Thank you for bringing this to our attention, these two articles along with 6 others were considered suitable for inclusion based on the inclusion criteria. However, they were subsequently excluded based on their quality rating for Richness. We have added an additional column to the Extended Data File 5 to identify these articles.

 9. Quality Assessment: In Figure 1, “records linked to included interventions (n=5)” and “relevant policy documents/guidelines (n=13)” seem to bypass the quality assessment, while they are partly assessed in Extended Data File 6 – please check for inconsistencies. Why is the quality assessment of the guidelines/policy documents “N/A”? What does mean the criterion “Richness at extraction”(in addition to “richness”) in Extended Data File 6?

 We did not feel the need to quality assess the policy or guideline documents as these are what is available to GPs to inform their practice and regardless of quality needed to be taken into account.

 The 27 articles included at full text review stage met the inclusion criteria, were assessed for relevance, rigour and richness, and scored either 3 or 4 on a four-point scale for richness. The five articles linked to articles already included were automatically included as they were regarded as having the same level of relevance and rigour as their linked articles and provided additional details which added to the richness of the data. These 32 articles were reassessed for richness at the extraction stage as more careful assessment was possible and it became obvious that the original score may have been too high for some articles as the data was extracted for each theory, the revised scores are available in Extended data file 6 under the heading “Richness at extraction”. Six of the articles were downgraded to a richness rating of 2, which means that they supplied limited data of interest, but were quick to extract it and could add weight to findings.

 10. Discussion of limitations and implications: the authors name a few limitations with regard to their methods. How do the authors assess the effect of their restrictions (e.g. publication language and period, quality feature "richness" as exclusion criterion) and limited resources (screening, data extraction and coding could mostly be done by only one person, the panel survey could not be tested a priori) on the results? Would there be a need for further research to support the results of the RRR?

 The restrictions you list are relevant to conventional systematic reviews but do not apply to the same extend to realist reviews. We have added some text to the introduction to highlight that the two methodologies are different. “ *A systematic review provides evidence in relation to the efficacy of an intervention, but does not identify why the intervention worked, or not. There are notable differences in the methodology of conventional systematic reviews and realist reviews. A realist review is iterative and non-linear and much more likely to make use of grey literature rather than relying solely on articles in academic journals. In addition, the evidence in a realist review is appraised to assess the weight of its contribution to refining the developed theories.”*

 It is not unusual for a realist review to be limited to the language of the researchers as all the nuances of the data need to be clearly understood. The restriction of the review to articles published between 2011 and 2021 was seen as a positive, ensuring the findings were relevant to current practice. The section on strengths and limitations does acknowledge that the time available to undertake this rapid realist review may have impacted on the range of articles included. Decisions regarding how to focus the review based on the time available again are an accepted process in realist reviews. We chose to limit the review to those articles with the richest data to get the most from the time available.

 The need to have two independent screeners for all the articles is not essential, as long as the main screener/extractor is knowledgeable about the topic of the realist review, has spent time with the rest of the research team and the panel of experts agreeing the theories in advance, which formed the basis of the extraction process and recorded in a transparent way the steps taken. However, we agree, this should be stated in the limitations. We had added a sentence to this effect *“The time limitations for this rapid review also meant that although two reviewers screened all relevant articles by title and abstract, there was only one reviewer for the full text reading of the articles, 10% of the excluded articles at this stage were reviewed by the 2nd reviewer. To reduce the potential subjective impact of this approach, team discussions, pilots and detailed inclusion and exclusion criteria guidance documents were developed, and a transparent record of the steps taken were recorded.”*

 Yes, there is a need for further research. The product of any realist review are theories based on available evidence, but are simply theories, which must be tested in future research to agree, refine or reject them. This would involve applying the theories to the design and implementation of an intervention and evaluating the results. We state this in the conclusions.

# Reviewer report: Reviewer response for version 1

**DOI:** 10.21956/hrbopenres.14833.r32547

**Published:** 2022-09-07

**Authors:** Cristin Ryan

**Affiliations:** 1 School of Pharmacy and Pharmaceutical Sciences, Trinity College Dublin, Dublin, Ireland

Thank you for the invitation to review this rapid realist review, which aims to identify and examine the contexts and mechanisms involved in outcomes relevant to the reduction of adverse drug events in community dwelling older adults in the general practice setting. This is a very important and complex topic, and one for which context is often overlooked.

 The paper represents a tremendous amount of work and is very well written. The background clearly outlines why the topic is important, and why a rapid realist review is the most appropriate methodology to use to provide answers to the research question.

 Robust realist methodology is used throughout, with input from stakeholders i.e. general practitioners to develop theories. The iterative nature of theory development is particularly valuable as the studies identified during the review were used to modify these as necessary.

 Results are presented clearly and logically. Guidance is provided for those wanting to develop interventions in general practice to reduce the occurrence of adverse drug events in community dwelling older adults. The results presented are contextualized for the Irish setting, but are of international relevance and importance. Indeed, the results presented could be extrapolated and applied to develop interventions in general practice focusing on other clinical areas and patient groups. The paper concludes that easily applied and well-resourced interventions, supported by national policy, could enhance the success of interventions aimed at reducing adverse drug events for community dwelling older adults in primary care in Ireland.

Are the rationale for, and objectives of, the Systematic Review clearly stated?

Yes

Is the statistical analysis and its interpretation appropriate?

Yes

Are sufficient details of the methods and analysis provided to allow replication by others?

Yes

Are the conclusions drawn adequately supported by the results presented in the review?

Yes

Reviewer Expertise:

Intervention development, theory, appropriate prescribing, polypharmacy

I confirm that I have read this submission and believe that I have an appropriate level of expertise to confirm that it is of an acceptable scientific standard.
